# Immunotherapy for high-grade endometrial stromal sarcoma: a narrative review bridging molecular insights to clinical translation

**DOI:** 10.3389/fimmu.2025.1645371

**Published:** 2025-09-04

**Authors:** Liubiqi Zhao, Yunyi Su1, Xiaoling Li, Fang Wang, Li He, Limei Fan, Lushuang Zhang

**Affiliations:** ^1^ Department of Obstetrics and Gynecology, Chengdu Women’s and Children’s Central Hospital, School of Medicine, University of Electronic Science and Technology of China, Chengdu, China; ^2^ Department of Obstetrics and Gynecology, Chongqing Medical University, Chongqing, China; ^3^ Department of Gynaecology and Obstetrics, the Second Hospital of Jilin University, Changchun, China

**Keywords:** high-grade endometrial stromal sarcoma, immunotherapy, TME, adoptive cell transfer, oncolytic virotherapy, PD-1/PD-L1, YWHAE-NUTM2

## Abstract

High-grade endometrial stromal sarcoma (HGESS) is a rare solid malignancy characterized by a poor prognosis and highly aggressive behavior. Currently, surgical resection remains the primary treatment for HGESS. Radiotherapy and chemotherapy can address local symptoms and enhance the quality of life of patients; however, they do not improve patient survival rates. Recent studies have found that the molecular characteristics of HGESS (such as gene fusions like YWHAE-NUTM2, ZC3H7B-BCOR, etc.) drive the high invasiveness of the tumor. Although immunotherapy has achieved significant breakthroughs in solid tumors, the immunosuppressive microenvironment of HGESS remains a key focus for future immunotherapy research. This narrative review comprehensively analyzes the interactions between alterations in the tumor microenvironment and immune escape mechanisms in HGESS. It also proposes a diverse range of immunotherapy options, including Oncolytic virus therapy, adoptive cell transfer therapy, immune checkpoint inhibitors, cancer vaccines, and combination strategies. We hold the view that a profound comprehension of the molecular immunological characteristics of HGESS, the identification of effective biomarkers, and the implementation of well-designed clinical studies are the indispensable routes to successfully translate immunotherapy into an effective treatment for this intractable HGESS.

## 1 Introduction

HGESS arises from endometrial stromal cells. According to the 2020 World Health Organization (WHO) tumor classification, it can be classified into four types: endometrial stromal nodules (ESN), low-grade endometrial stromal sarcoma (LGESS), HGESS, and undifferentiated uterine sarcoma (UUS). LG-ESS is the most prevalent subtype. It exhibits slow growth but may have a late recurrence (1). UUS is characterized by extremely high malignancy, lacks differentiation features, and has a very poor prognosis. HGESS is a highly malignant solid tumor with extremely poor prognosis and rare biological behavior, characterized by high local invasiveness and a high recurrence rate. The clinical manifestations of HGESS include irregular vaginal bleeding, pelvic pain, and mass. These early symptoms may not be obvious or specific. As a result, HGESS often reaches an advanced stage by the time of diagnosis. These characteristics also lead to the poor prognosis of this disease, with a median overall survival of 11 to 23 months (2) and a mortality rate as high as 70.6% (3).

High-grade endometrial stromal sarcoma (HGESS) poses challenges in clinical diagnosis due to its subtle early symptoms. Accurate staging of HGESS relies on a comprehensive evaluation using multimodal imaging techniques. Among these, magnetic resonance imaging (MRI) is the preferred modality (4). It can precisely depict the depth of myometrial invasion by the primary tumor, parametrial invasion, and cervical involvement. Characteristic findings on MRI include heterogeneous high signal intensity on T2-weighted images, early enhancement in dynamic contrast-enhanced imaging, and high signal on diffusion-weighted imaging (DWI) with decreased apparent diffusion coefficient (ADC) values (5). Computed tomography (CT) can rapidly screen for lung or liver metastases and lymph node enlargement. The typical CT features are an inhomogeneous uterine mass with necrotic areas and mild enhancement. Functional metabolic imaging, such as ^18^F-fluorodeoxyglucose positron emission tomography/computed tomography (^18^F-FDG PET/CT) (6), exhibits a sensitivity of >90% for high-risk endometrial stromal sarcoma (ESS). It can detect early lymph node or bone metastases through increased metabolic activity (elevated standardized uptake value maximum, SUVmax). Moreover, ^18^F-FDG PET/CT plays a unique role in monitoring residual or recurrent lesions after treatment, as metabolic changes occur earlier than morphological alterations (7).The combined use of these three imaging methods significantly enhances the timeliness and accuracy of diagnosis, providing crucial support for precise clinical diagnosis and treatment planning.

The etiology of HGESS remains not fully elucidated. Many scholars postulate that chromosome rearrangement might be the underlying cause of this disease. Initial research has indicated that HGESS patients with the t(10; 17) (q22; p13) translocation exhibit the YWHAE-NUTM2 gene fusion. This fusion subsequently leads to the ZC3HGB-BCOR gene fusion or tandem duplication within BCOR, thereby contributing to the high-grade morphological features and aggressive clinical behavior of the disease (8–10). Additionally, other rare gene rearrangements, such as EPC1-SUZ12, EPC1-BCOR, CREBBP-BCOR, LPP/BCOR, and BCOR ITD, have also been reported to be associated with the development of this disease (11–13). The most prevalent YWHAE-NUTM2 fusion in our research involves the combination of YWHAE (14-3-3ϵ) and NUTM2 (a member of the NUT family), which disrupts normal transcriptional regulation. This disruption may generate a fusion protein containing the breakpoints of the two genes. The distinctive amino acid sequence near the breakpoint may be presented as a neoantigen by Major histocompatibility complex-I(MHC-I )molecules. *In vitro* experiments have demonstrated the presence of CD8+ T cell clones in the peripheral blood of patients that can recognize the fusion peptide (9).

Currently, surgical treatment serves as the primary approach for treating HGESS. This includes total hysterectomy, bilateral salpingo-oophorectomy. For patients with stage II or more advanced HGESS, adjuvant treatment with systemic therapy, with or without radiotherapy, should be administered. Given the variations in the diagnosis timing of HGESS and the different disease severities, the treatment plan should be individually evaluated. Postoperative radiotherapy enhances locoregional control but does not improve overall survival. For newly diagnosed metastatic HGESS, when symptom relief cannot be easily achieved through systemic therapy and/or surgical resection, palliative radiotherapy can be carried out to alleviate local symptoms and improve the quality of life. Since tumors often exhibit extensive invasion and metastasis, it is challenging to obtain negative surgical margins, and radiotherapy cannot increase the overall survival rate (14). Thus, an increasing number of studies suggest that genetic testing, attempting individualized immunotherapy, and encouraging patients to participate in clinical trials are advisable for patients with late recurrence and those who have failed conventional treatment.

The initiation stage of cancer is triggered by DNA damage induced by genetic mutations, chemical carcinogens, radiation, and viral infections. This damage upregulates oncogenes and downregulates tumor suppressor genes, thereby leading to the transformation of initiator cells. Subsequently, growth factors and survival signals activate oncogenes and promote tumor formation by inducing enhanced cell proliferation of initiator cells. Moreover, the uncontrolled growth during this process significantly raises the probability of mutation. The immune system plays a dual role in suppressing tumor growth, which can be characterized by three processes: elimination, equilibrium, and escape. During the elimination phase, the immune system effectively recognizes and eliminates nascent tumor cells. Most abnormal cells are eradicated at this stage, remaining asymptomatic and undetectable (15). In the equilibrium phase, the immune system engages in a “protracted battle” with tumor cells. The few cancer cells that survive the elimination phase experience repeated cycles of proliferation and elimination, enabling them to enter the equilibrium phase and persist without tumor formation (16). This phase represents a crucial window for immunotherapy interventions (for example, Programmed death-1(PD-1) inhibitors may disrupt the balance). During the escape phase, tumor cells breach the immune defense line, grow uncontrollably, and metastasize. Detectable tumors form, accompanied by invasive metastases (such as brain metastases from lung cancer) (17).Typically, at this stage, we can utilize Immune checkpoint inhibitors(ICIs) (such as anti-PD-1) to reverse T-cell suppression, employ Chimeric antigen receptor T cells (CAR-T) therapy to target specific antigens, or use combined chemotherapy/radiotherapy to break down the immunosuppressive barrier.

**Figure 1:**
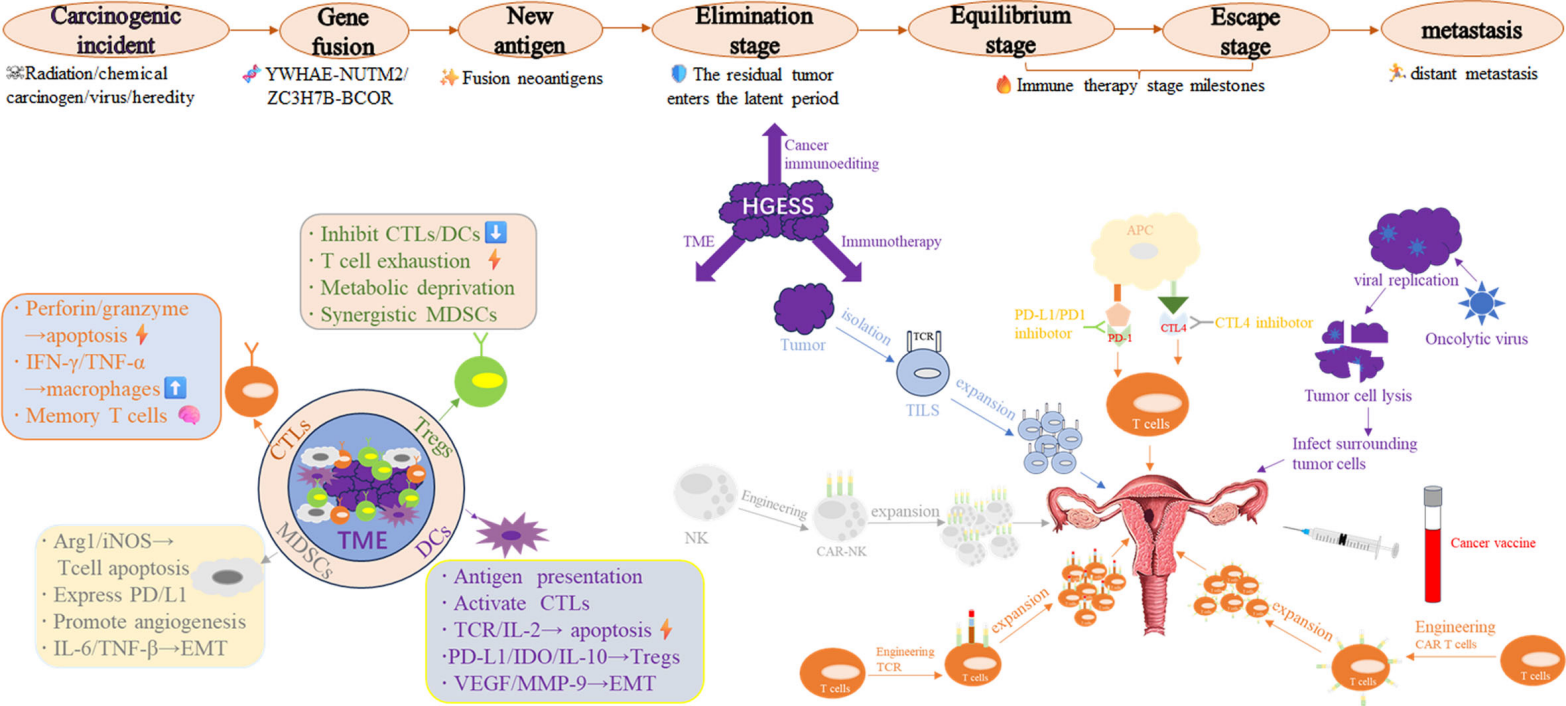
Integrated schematic diagram of the HGESS immune microenvironment and immunotherapy strategies.

This article undertakes a comprehensive analysis of tumor microenvironment (TME) in HGESS. It elaborates on a diverse range of immunotherapies within the framework of current research. For instance, OVT, cancer vaccines, adoptive cell transfer therapies (including T cell receptor (TCR) engineered T cell therapy, CAR-T cell therapy, CAR-NK cell therapy, and tumor cell infiltrating lymphocytes (TILs)), and immune checkpoint inhibitors (ICIs) are currently under development. The present challenges and future exploration directions are also summarized (**Figure 1**).

## 2 Immune microenvironment of HGESS

Understanding the tumor microenvironment is crucial for the success or failure of immunotherapy. The tumor microenvironment is a complex and dynamic system consisting of various immune cells and non-cellular components that interact with one another, influencing tumor progression, metastasis, and treatment response. Each type of tumor exhibits distinct characteristics and mechanisms of action. The TME is a complex niche where cancerous and non-cancerous cellular components develop. Its immune status can be classified as “cold” or “hot” based on the production of proinflammatory cytokines and the level of immune cell infiltration. A “cold” TME is characterized by a lower number of inflammatory immune cells, an immunosuppressive environment, a poor prognosis, and an inadequate response to immunotherapy. Conversely, a “hot” TME is associated with activated immune cells and has a higher response rate (18–20).Intrinsic immune cells, including NK cells, eosinophils, basophils, and phagocytes such as mast cells, neutrophils, monocytes, macrophages, and DCs, play a role in tumor suppression by directly killing tumor cells or triggering adaptive immune responses. The adaptive immune system consists of lymphocytes, including B cells, which play a crucial role in humoral immune responses, and T cells, which are involved in cell-mediated immune responses (21–24). Some scholars have demonstrated that the degree of immune infiltration in HGESS ranges from moderate to high. This is mainly characterized by the infiltration of central memory CD4 T cells, myeloid-derived suppressor cells (MDSC), central memory CD8 T cells, plasmacytoid dendritic cells, effector memory CD8 T cells, regulatory T cells, and T follicular helper cells (25). Although studies have indicated that the infiltration of CD8+ T cells in Chengdu is relatively high, most CD8+ T cells are in a state of functional exhaustion due to the high expression of inhibitory receptors such as PD-1 and TIM-3 (9). In the following sections, we will primarily focus on these cells to discuss their roles in the immune microenvironment.

### 2.1 The role of T lymphocytes in the tumor microenvironment

In combating tumor cells, CTLs serve as primary players. They directly induce tumor cell apoptosis by releasing perforin and granzyme B, while concurrently secreting cytokines such as interferon-gamma(IFN-γ) and TNF-α to inhibit tumor proliferation and activate macrophages. Furthermore, CTLs generate long-lived memory cells, providing long-term immunity against future cancer encounters (26). This specific recognition is accomplished through the interaction between the TCR on CTLs and the peptid-MHC (major histocompatibility complex) on the surface of tumor cells. Once recognition takes place, CTLs induce target cell death via apoptosis. The dysfunction and exhaustion of CTLs in tumors are characterized by a diminished capacity to carry out their predefined functions and the presence of inhibitory receptors such as PD-1, T cell immunoglobulin and mucin domain 3 (TIM-3), and lymphocyte activation gene 3 (LAG-3), which hinder their activity. Additionally, their gene expression patterns also undergo changes. According to a model for studying pancreatic cancer, signaling at the IL-18 receptor is responsible for regulating the exhaustion of tumor-targeting CD8+ T lymphocytes. This is achieved through the activation of the IL-2/STAT5/mTOR pathway (27).

Tregs, the “messengers of peace” within the immune system, are specifically tasked with suppressing excessive immune responses. They play a crucial role in maintaining immune balance, preventing the immune system from attacking the body’s own tissues or triggering excessive inflammation. Tregs can prevent autoimmune diseases, limit chronic inflammatory disorders, and uphold peripheral tolerance. Moreover, Tregs are of great significance in the tumor microenvironment, influencing cancer progression and immune responses (35). Tregs are involved in suppressing immune responses through several mechanisms: (1) They initiate a tolerogenic environment by inducing tolerogenic DCs. These DCs then promote T-cell exhaustion and the expression of a major transcription factor known as forkhead box P3 (FOXP3). (2) Cytotoxic T-lymphocyte-associated antigen 4(CTLA-4) binds to CD80/CD86 on APCs, thereby inhibiting the activation of other T cells by APCs. Simultaneously, Tregs can secrete granzyme or induce apoptosis of effector T cells via the Fas/FasL pathway. Additionally, the secretion of inhibitory cytokines, such as Transforming growth factor-beta(TGF-β), IL-10, and IL-35, inhibits the production of inflammatory factors, the proliferation of T cells, and the activity of macrophages. (3) Tregs highly express IL-2 receptors like CD4 and CD25, consuming large amounts of IL-2. Alternatively, by inhibiting the adenosine pathway or tryptophan metabolism in Tregs, they can disrupt the activity of effector T cells through metabolic alterations. This also leads to the removal of the inhibitory effect of Tregs on effector T cells. Currently, we can surmount the immunosuppression of the tumor environment by combining PD-1 inhibitors with TreGs-targeting drugs to address the abovementioned characteristics (28, 29).

### 2.2 The role of myeloid-derived suppressor cells in the tumor microenvironment

MDSCs are a group of immature myeloid cells typically classified into two types: monocyte-derived (M-MDSC) and granulocyte-derived (polymorphonuclear cell-derived [PMN]-MDSC). M-MDSC closely resembles monocytes in terms of phenotype and physical characteristics, while PMN-MDSC is equivalent to neutrophils (30). MDSCs represent a crucial immunosuppressive cell population within the TME. They facilitate tumor immune escape, angiogenesis, metastasis, and treatment resistance through multiple mechanisms: (1) Suppression of anti-tumor immune responses: MDSCs deplete arginine in the microenvironment via Arg1 and iNOS, thereby inhibiting the expression of the T cell CD3ζ chain and suppressing T cell activation and proliferation. They directly damage the TCR and IL-2 signaling pathways and induce apoptosis in T cells. Some MDSCs express PD-L1, which inhibits T cell function through the PD-1/PD-L1 pathway (31). Additionally, they downregulate NK cell-activated receptors (such as NKG2D), inhibiting IFN-γ secretion and cytotoxicity. Moreover, they impede dendritic cell (DC) maturation, hinder antigen presentation, and impair T-cell priming (32, 33). (2) Facilitate tumor angiogenesis: The secretion of factors such as Vascular endothelial growth factor(VEGF), bFGF, and MMP9 promotes endothelial cell proliferation and angiogenesis. IL-10 can induce the pro-angiogenic M2 polarization of tumor-associated macrophages (TAMs). (3) Drive tumor cell metastasis: By secreting IL-6 and TGF-β, it induces epithelial-mesenchymal transition (EMT) and enhances tumor cell invasion. Formation of the pre-metastatic microenvironment: MDSCs accumulate in metastatic target organs (such as the lung and liver) and suppress local immune surveillance. (4) Mediate treatment resistance: The release of the S100A8/A9 protein activates the NF-κB pathway to promote tumor cell survival. It protects tumor cells by scavenging free radicals generated by radiotherapy. It counteracts the efficacy of PD-1 antibodies or CAR-T cells.

MDSCs interact within the TME mainly through the following mechanisms: (1)Recruitment signals: Tumor cells and stromal cells secrete factors such as granulocyte-macrophage colony-stimulating factor (GM-CSF), interleukin-6 (IL-6), VEGF, and chemokine (C-C motif) ligand 2 (CCL2) to recruit MDSCs into the TME (34–36). (2) Metabolic reprogramming: Hypoxia and lactate in the tumor microenvironment induce the immunosuppressive function of MDSCs via hypoxia-inducible factor-1α (HIF-1α). Fatty acid oxidation (FAO) enhances the survival and inhibitory activity of MDSCs. (3) Synergism with other immunosuppressive cells: Interaction with Tregs: MDSCs induce the expansion of Tregs through TGF-β and interleukin-10 (IL-10). MDSCs interact with tumor-associated macrophages (TAMs) to form an immunosuppressive network and promote tumor progression. MDSCs are the core components of the “immunosuppressive network” in the tumor microenvironment. By inhibiting the immune response and promoting angiogenesis and metastasis, MDSCs act as “accomplices” in tumor progression. Treating MDSCs requires considering their heterogeneity and dynamic regulatory mechanisms, in combination with chemotherapy, targeted therapy, and immune checkpoint inhibitors (ICIs). This approach can ultimately reshape the anti-tumor immune microenvironment, offering a promising strategy for the future treatment of HGESS.

### 2.3 The role of dendritic cells in the tumor microenvironment

DCs can be classified into classical DCs, plasmacytoid DCs, and regulatory DCs. They serve as the “sentinels” and “commanders” of the immune system, playing a complex and crucial dual role in the TME. On one hand, they can activate the anti-tumor immune response; on the other hand, they may be “coopted” by tumors and become facilitators of immunosuppression. The following details the specific roles and mechanisms of DCs in the TME.

“The “anti-tumor” effects of DCs in the tumor microenvironment are as follows: (1) Antigen presentation and T cell activation: DCs can engulf antigens (such as neoantigens) released from tumor cell debris and dead cells. They present these antigens to CD8+ T cells via major histocompatibility complex (MHC) class I molecules and initiate CTLs. DCs express molecules like CD80/CD86 and CD40, providing a second signal for T cell activation (37, 38). (2) Recruitment and coordination of immune cells: DCs secrete chemokines (such as CXCL9/10), which attract CTLs and NK cells to infiltrate tumors. Additionally, through interleukin-12 (IL-12), DCs promote the differentiation of Th1 cells and CTLs into effector cells. (3) Direct killing of tumor cells: Some DCs express tumor necrosis factor-related apoptosis-inducing ligand (TRAIL) or perforin, directly inducing tumor cell apoptosis.”

The “Pro-Tumor” Role of Dendritic Cells (DCs) in the Tumor Microenvironment: (1) Functional Inhibition and Phenotypic Aberration: Tumors secrete factors such as VEGF, interleukin-10 (IL-10), and TGF-β. These factors impede the maturation of DCs, leading to low expression of major histocompatibility complex class II (MHC II) and costimulatory molecules. Additionally, lactate accumulation in the TME inhibits the antigen presentation ability of DCs (31). (2) Induction of Immune Tolerance: Immature DCs induce the generation of Tregs via programmed death-ligand 1 (PD-L1), indoleamine 2,3-dioxygenase (IDO), and IL-10. Plasmacytoid DCs (pDCs) secrete TGF-β and thymic stromal lymphopoietin (TSLP), which promote a T helper 2 (Th2) bias and immunosuppression. (3) DCs Promote Angiogenesis and Metastasis: DCs secrete VEGF and matrix metalloproteinase-9 (MMP-9), which directly support tumor angiogenesis. They also induce EMT and enhance the invasive ability of tumor cells.

The mechanism of DC “acclimatization” within the tumor microenvironment is as follows: (1) Cytokine storm: GM-CSF and IL-6 induce the differentiation of myeloid precursors into tolerogenic DCs rather than effector DCs. (2) Exosome regulation: Tumor exosomes carry PD-L1 and miRNAs (such as miR-21-5p), which inhibit the function of DCs. (3) Metabolic hijacking: Tumor cells compete for glucose consumption, resulting in energy depletion in DCs (manifested as an AMPK/mTOR imbalance).

Based on the above-mentioned mechanism of action, the following approaches can be employed for anti-tumor therapy: (1) Develop DCs vaccines. Load tumor antigens (such as neoantigens and tumor lysates) *in vitro* to activate anti-tumor T cells, and then reinfuse them. (2) Reverse the PD-L1-mediated T cell suppression on DCs. This can be achieved by combining anti-PD-1/PD-L1 antibodies with immune checkpoint inhibitors (ICIs). Alternatively, use anti-CD40 agonist antibodies (such as Selicrelumab) to activate DCs and enhance antigen presentation. (3) Implement targeted metabolic reprogramming. Block tryptophan depletion and restore DCs activity by inhibiting indoleamine 2, 3-dioxygenase (IDO). Alternatively, supplement L-arginine or block arginase to enhance the function of DCs. (4) Engineer DCs genetically. Enhance the ability of DCs to migrate to lymph nodes by introducing chemokine receptors (such as CCR7). Express cytokines (such as IL-12, IFN-α) to boost immune activation. As stated above, overcoming the functional inhibition of the TME on DCs is crucial for improving the efficacy of cancer immunotherapy, including vaccines and CAR-T cells. Future treatments should integrate specific regulation of DCs subsets, metabolic intervention, and combination therapy to reshape the “equilibrium” of the immune microenvironment in the direction of anti-tumor.

## 3 Immunotherapy

Immunotherapy, which endeavors to boost the body’s natural defenses to eliminate cancer cells, represents a significant breakthrough in cancer treatment and has transformed the field of oncology. Cancer is a genomic disorder characterized by genomic instability during tumor progression. This instability is manifested by the accumulation of numerous point mutations and structural alterations. These genomic changes can give rise to tumor antigens that the immune system can recognize as foreign, thereby triggering cellular immune responses (39, 40). The immune system plays a crucial role in immune surveillance (41, 42). Immune cells from both the adaptive and innate immune systems infiltrate the TME to regulate tumor progression (43, 44). Although HGESS exhibit substantial immune infiltration, the functionally inhibitory microenvironment restricts the therapeutic response. Targeted intervention strategies are explored below. Currently, immunotherapies can be classified into the following categories: tumor-lytic virotherapy, cancer vaccines, cytokine therapy, adoptive cell transfer, and immune checkpoint inhibitors (ICLs) (45).

### 3.1 Oncolytic virus therapy

Oncolytic virus therapy, which utilizes bacterial or viral infection to boost the immune response against cancer, entails infecting tumor cells with genetically engineered viruses. This process stimulates a proinflammatory environment to enhance systemic antitumor immunity while sparing normal cells (46, 47). Through a dual killing mechanism. The first is targeted lysis: Oncolytic viruses (OVs) selectively infect tumor cells and directly lyse these cells via virus replication. For instance, the Myxoma virus targets tumor cells that overexpress ribonucleotide reductase in canine soft tissue sarcomas (48). The second is to exert its effect through immune activation: releasing tumor antigens, activating dendritic cells (DCs), and recruiting T cells. For example, the Maraba virus promotes the infiltration of CD8^+^ T cells in the sarcoma microenvironment (49). Carrying immunomodulatory genes (such as IL-12, CXCL10) enhances anti-tumor immunity. For example, an oncolytic adenovirus carrying CXCL10 significantly improves the efficacy of PD-1 inhibitors (50, 51). H101, a genetically engineered oncolytic adenovirus, was approved in China for the treatment of nasopharyngeal carcinoma in November 2005 (52). In 2015, the U.S. Food and Drug Administration (FDA) approved the first oncolytic virus (T-VEC) for the treatment of metastatic melanoma (53). T-VEC is an engineered virus based on attenuated herpes simplex virus type 1 (HSV-1). In a phase Ib clinical trial of T-VEC combined with the anti-PD-1 antibody (pembrolizumab), patients with metastatic melanoma exhibited an overall response rate of 62% and a complete response rate of 33% (NCT04068181) (54). In 2021, Japan approved the world’s first oncolytic virus for the treatment of malignant gliomas (55). In a randomized phase III trial involving 160 patients with advanced squamous cell carcinoma of the head, neck, or esophagus, those treated with cisplatin/5-FU in combination with H101 had a response rate that was 78.8% higher, compared to 39.6% for patients treated with cisplatin/5-FU alone (56). In the realm of soft tissue sarcomas, research has demonstrated that virus can significantly impede the growth of *in-situ* soft tissue sarcomas, prolonging the survival period by 40% (57). The third-generation herpes simplex virus type 1 (G47Δ) exhibits strong penetrability against drug-resistant soft tissue sarcomas and yields obvious therapeutic outcomes. The Myxoma virus shows good tolerance in canine soft tissue sarcomas, only inducing transient fever (48, 58). The oncolytic vaccinia virus reduces the volume of 63% of canine sarcomas by over 50 (57). Moreover, it can trigger immune remodeling of the tumor microenvironment in advanced solid tumors, including sarcomas, with a disease control rate of 32% (51).

Oncolytic viruses (OVs) possess significant anti-tumor potential and also serve as powerful partners for other immunotherapies. OVs can function as radiosensitizers, inducing DNA damage and promoting viral proliferation (59, 60). Simultaneously, chemotherapy can enhance oncolytic virotherapy by enabling OVs to evade antiviral immune responses. Cyclophosphamide (CPA), a chemoalkylating agent used as an immunosuppressive drug, can induce tumor cell apoptosis when combined with oncolytic virotherapy. Moreover, when combined with ICIs, CAR-T cells, and epigenetic drugs, it exhibits a synergistic anti-tumor effect. In a phase Ib trial, T-VEC in combination with the anti-PD-1 antibody pembrolizumab demonstrated encouraging antitumor activity in patients with stage IIIB and IV melanoma. This combination altered the TME, increased the infiltration of cytotoxic CD8+ T cells into the tumor site, and elevated interferon gamma (IFNγ) levels (54). The AK inhibitor Oclacitinib boosts the replication efficiency of Myxoma virus in canine high-grade sarcomas (61). Trabectedin facilitates the replication and immune effector functions of OV in osteosarcoma (62). Park et al. engineered an oncolytic vaccinia virus (VACV) carrying CD19, a naturally occurring antigen, enabling tumor cells to be targeted by CD19-specific CAR-T cells. More copies of the virus are released from dying tumor cells, spreading CD19 expression to neighboring tumor cells and eliciting a more effective antitumor response (63).

Virotherapy for ovarian cancer (OV) is an emerging form of cancer immunotherapy. A more in-depth comprehension of the interactions among oncolytic viruses (OV), immune cells, tumor cells, and other components of the TME will expedite the development of more innovative oncolytic viruses and ultimately result in improved clinical outcomes for patients (64). Based on current research, we have grounds to believe that in the future anti-tumor research domain of HGESS, we can also incorporate oncolytic virus treatment, continue to develop such novel drugs, and combine them with other immunotherapy approaches to achieve a better anti-tumor effect. The safety of large animal models has been confirmed, and the potential for immune activation has been validated in early clinical trials. The core challenges primarily center on enhancing delivery efficiency, deciphering drug resistance mechanisms, and optimizing individualized treatment regimens. Current ongoing clinical studies encompass: the treatment of osteosarcoma and advanced solid tumors using OV(R130) (NCT06171282, NCT05851456, NCT05961111), as well as the exploration of the safety and efficacy of REOLYSIN^®^ (a therapeutic reovirus) for metastatic soft tissue sarcoma (NCT00503295). We are keenly awaiting the outcomes of these studies. With the innovation of vector engineering (such as promoter design and armed gene regulation) and combination strategies, oncolytic viruses (OVs) are anticipated to emerge as a crucial pillar of precision immunotherapy for sarcoma (65–67).

### 3.2 Cancer vaccines

Virotherapy for ovarian cancer (OV) represents an emerging modality of cancer immunotherapy. A deeper understanding of the interactions among oncolytic viruses (OV), immune cells, tumor cells, and other elements of the TME will accelerate the development of more innovative oncolytic viruses and ultimately lead to enhanced clinical outcomes for patients (68). Based on current research, we have reason to believe that in the future anti-tumor research of HGESS, oncolytic virus treatment can also be incorporated. We can continue to develop such novel drugs and combine them with other immunotherapy methods to achieve a better anti-tumor effect.

The antigenic targets of cancer vaccines are the core of their anti-tumor effects. Based on antigens, they are primarily classified into two categories: tumor-specific antigens and tumor-associated antigens (TAAs). Tumor-specific antigen targets are novel antigens generated by gene mutations in tumor cells. These antigens are exclusively expressed in tumor cells. They can be highly individualized, evade autoimmune attacks (since they are not expressed in normal tissues), and possess strong immunogenicity, which can activate the CD8+ T cell response. Such a target demands sufficient cancer specificity, a peripheral repertoire of non-autoantigen species with functional antigen-specific T cells that can be expanded upon antigen delivery, and immunodominance to maximize the induction of immune responses against cancer cells. Tumor-associated antigen targets mainly encompass molecular antigens, overexpressed antigens, carbohydrate antigens, etc. In 1998, the gp100 vaccine, which targeted melanoma marker antigens in combination with IL-2, achieved an ORR of up to 16% (69). In 2010, the first US Food and Drug Administration (FDA)-approved Sipuleucel-T (for prostate cancer) dendritic cell (DC) vaccine, when combined with a CTLA-4 inhibitor, could extend the survival time by 8.5 months (70). In 2014, the breast cancer vaccine NeuVax (E75 peptide) combined with trastuzumab enhanced the 5-year disease-free survival rate to 89% (compared with 72%) (71). Karbach J et al. demonstrated in their research(NCT00623831)that cancer vaccines can mediate tumor regression via immune mechanisms in various tumors, including sarcomas, and extend the overall survival time (72). Clinical trials of cancer vaccines for sarcoma (NCT00005628, NCT01241162, NCT00069940, NCT03357315, NCT01803152) are currently in progress. We anticipate achieving positive research outcomes. Although the aforementioned vaccines are effective, their clinical efficacy remains limited. Thus, the key bottleneck restricting the development of cancer vaccines lies in how to rapidly and comprehensively identify the best cancer antigen capabilities.

In the current advancement of cancer vaccines, the combination of the nanoparticle delivery system and mRNA vaccine technology exhibits remarkable advantages. These two technologies overcome the limitations of traditional vaccines through complementary mechanisms and have emerged as a significant breakthrough in the field of immunotherapy. Thanks to the flexibility of the target, mRNA vaccines can rapidly encode any antigen without the need for complex protein purification processes. Moreover, the *in vitro* transcription technology is well-established, enabling large-scale production. Once mRNA enters cells, it is directly translated into antigens with native conformations, which are presented via the MHC-I and MHC-II pathways, simultaneously activating CD8+ T cells and CD4+ T cells. Additionally, mRNA vaccines have notable advantages in terms of safety. Since mRNA does not enter the nucleus, it avoids the risk of insertion mutations, and its half-life can be controlled, reducing the risk of autoimmune reactions. Research has demonstrated that nanoparticle delivery systems can safeguard the stability of mRNA and protect it from degradation by ribonuclease in the blood (73). Targeted delivery to lymph nodes or APCs by surface modifications (e.g., mannose, targeted peptides) also facilitates mRNA release into the cytoplasm by ph-sensitive lipids or cationic polymers (74). At present, studies have shown that the ORR of the mRNA vaccine designed for KRAS G12D mutation in colorectal cancer patients combined with PD-1 inhibitors can reach 50% (75). The mRNA-4157 vaccine combined with pembrolizumab (Keytruda) reduced recurrence or mortality by 44% and improved 18-month recurrence-free survival to 83.4% in surgically resected stage III/IV melanoma. The combination of nanoparticle delivery systems with mRNA vaccines significantly enhances the therapeutic potential of cancer vaccines through efficient delivery, precise immune activation, and flexible design. Currently, there are no relevant tests for HGESS anti-tumor cancer vaccines. Nevertheless, with the advancement of delivery technologies and antigen screening algorithms, such vaccines are anticipated to become a fundamental approach for the immunotherapy of solid tumors. This offers significant inspiration for our future research on HGESS anti-tumor immunotherapy.

### 3.3 Adoptive cell transfer therapy

Adoptive cell transfer therapy utilizes autologous immune cells that are isolated *in vitro* or genetically engineered for expansion and subsequently reinfused into the patient to eliminate cancer cells. This therapy has demonstrated durable clinical effectiveness. The essence of this approach lies in the use of genetic engineering or *in vitro* activation to confer enhanced tumor-recognition and killing abilities to immune cells, especially in hematologic and solid tumors (76–78). The main current therapy types include TILs chimeric antigen receptor (CAR)-T cell therapy, T-cell receptor-engineered T cells(TCR-T) cell therapy, and NK cell therapy.

Tumor-infiltrating lymphocytes (TILs) originate from T cells with natural anti-tumor activity isolated from tumor tissues. These cells are expanded *in vitro* and then reinfused with the support of adjuvant cytokines. In a recent phase III trial (NCT02360579) involving 178 patients with advanced melanoma who had not responded to PD-1 inhibitor therapy, the use of LN-144 (Lifileucel) led to an ORR of 36% and significantly prolonged survival. Currently, the US Food and Drug Administration (FDA) has approved this approach for the treatment of advanced melanoma (79). In a phase II trial (NCT03108495) involving 27 patients with recurrent/metastatic non-small cell lung cancer (mNSCLC), LN-145 (Lifileucel) demonstrated an ORR of 44% (80). At present, researchers are carrying out a Phase 1, multi-center, open-label study (NCT06566092). This study employs a two-stage (dose escalation and dose expansion), single-arm design. Its aim is to assess the safety and tolerability of autologous tumor-infiltrating lymphocytes (TILs) infusion in patients with various tumor types, including sarcoma. The advantage of this TILs therapy is that it does not necessitate genetic modification. It naturally targets individual antigens and reduces the risk of off-target effects. However, immunosuppressive factors in the TME, such as TGF-β and PD-L1, cause TILs to become exhausted. To address these limitations, TILs can be enhanced through *in vitro* amplification techniques. Additionally, genetic engineering of TILs can be carried out by knocking out PD-1 or overexpressing chemokines. Current studies have also indicated that TILs can be used in combination with PD-1/CTLA-4 inhibitors (81).

CAR-T cells are genetically engineered T lymphocytes. These cells express a synthetic receptor that can recognize antigens on the surface of tumors and trigger T cells to kill tumor cells. Currently, CAR-T cell therapy has demonstrated a significant therapeutic effect in hematological tumors. Six types of CAR-T cell therapies have been approved by the US FDA for the treatment of hematological malignancies. However, due to antigen heterogeneity and the immunosuppressive microenvironment, there are substantial challenges in treating solid tumors. An open-label, non-randomized, multicenter Phase I/II trial (NCT07066982) is assessing the safety and efficacy of autologous dual-target CAR-T cells (CD146/HER2) in around 40 patients with relapsed/refractory advanced sarcoma (including both children and adults). The study is divided into two phases. In Phase I, dose escalation is employed to determine the safe dose (primary endpoint: dose-limiting toxicity). In Phase II, an expanded cohort is utilized to evaluate the preliminary efficacy (objective response rate, progression-free survival, overall survival). All patients will receive lymphodepleting chemotherapy (cyclophosphamide + fludarabine) followed by sequential infusion of dual CAR-T cells and will be followed up for 36 months to monitor long-term safety. With the optimization of CAR-T cell therapy technology, such as the development of dual-target CAR-T to enhance tumor targeting, the conversion of the PD-1 signal into a costimulatory signal, the modification of chemokine receptors, and metabolic regulation to promote cell infiltration and persistence, breakthroughs have been achieved in the treatment of solid tumors (82–87).

TCR-T cell therapy represents an individualized immunotherapy approach. It involves genetically engineering the antigen recognition TCR of T cells to specifically target antigens within tumor cells. Distinct from CAR-T, TCR-T can recognize peptide antigens presented by MHC molecules. This breakthrough overcomes the limitation of CAR-T, which can only target surface antigens, and thus demonstrates unique advantages in the treatment of solid tumors. Current studies have indicated that TCR-T therapy can significantly improve the ORR in synovial sarcoma, ovarian cancer, lung cancer, pancreatic cancer, and even cervical cancer, with some patients achieving a lasting remission of over 12 months (88, 89). Gyurdieva et al. demonstrated that letetresgene autoleucel (lete-cel) - an autologous T-cell therapy expressing high-affinity T-cell receptors (TCRs) specific for NY-ESO-1 - achieved a 50% objective response rate in metastatic synovial sarcoma patients (NCT01343043). *Post-hoc* analyses identified key response biomarkers: elevated baseline IL-15 levels (*p*=0.011) and higher infusion doses of transduced effector memory CD8+ T cells (*p*=0.039). Responders exhibited significantly increased IFNγ, IL-6, and peak T-cell expansion post-infusion (*p* < 0.01). Tumor microenvironment assessment revealed lete-cel infiltration correlated with downregulated macrophage-associated gene expression, suggesting immune remodeling. This study provides potential predictive biomarkers for optimizing lymphodepleting chemotherapy (LDR) and cellular dosing strategies (90). Multiple ongoing clinical trials (NCT05620693, NCT06083883, NCT06942143, NCT06889766, NCT05296564, NCT03250325) are further evaluating NY-ESO-1-targeted TCR-T cell therapies in advanced sarcomas. Regarding the current core challenges of TCR-T therapy, such as off-target toxicity, antigen escape, and difficulties in neoantigen screening, in the future, we can utilize AI to predict individualized neoantigens. Additionally, gene editing technology can be employed to knockout endogenous TCR and PD-1, thereby enhancing the competitive advantages of the modified TCR. With its ability to target intracellular antigens and strong adaptability to solid tumors, TCR-T has emerged as the focus of the next generation of cell therapy following CAR-T. With the breakthroughs in AI-assisted target screening and gene editing technology, TCR-T is anticipated to achieve clinical translation in our HGESS, propelling precision immunotherapy to a new stage.

NK cell therapy is a tumor immunotherapy approach grounded in natural immune cells. This therapy can enhance the ability of NK cells to recognize and kill tumor cells through activation or genetic engineering. Compared with T cell therapy, NK cells offer advantages such as no requirement for antigen presentation, no risk of graft-versus-host disease (GVHD), and allogeneic versatility. As a result, it is emerging as a promising field for the treatment of solid tumors. However, the survival time of NK cells is typically less than one month. Vascular barriers and matrix obstacles limit their efficacy. Additionally, large-scale production is costly, and quality control is challenging. These factors increase the difficulty of clinical translation and also represent the directions for future efforts. With the breakthroughs in genetic engineering and stem cell technology, genetically engineered NK cells (CAR-NK) and stem cell-derived NK cells (iPSC-NK) are anticipated to become the “star” main therapies for solid tumors in the future, propelling tumor treatment into an era of universal treatment.

### 3.4 Immune checkpoint inhibitors

However, cancer cells frequently exploit these checkpoints to evade immune surveillance. ICIs have become one of the most crucial immunotherapies. By interrupting co-inhibitory signaling pathways, ICIs reactivate antitumor immune responses and facilitate immune-mediated elimination of malignant cells (39, 91). ICIs block immunosuppressive receptors, including CTLA-4 (cytotoxic T-lymphocyte-associated antigen 4), PD-1 (programmed death 1), and their ligand PD-L1 (programmed death ligand 1). Meanwhile, they function as antitumor agents by modifying the TME of tumors.

CTLA-4 is a co-inhibitory molecule expressed on T cells, and its function is to negatively regulate T-cell activation. A groundbreaking study demonstrated that blocking CTLA-4 with antibodies could induce a potent immune response and result in tumor shrinkage, thereby inaugurating the era of using antibodies to disinhibit immune cells for enhancing antitumor immune responses (92–94).Following clinical trials and efficacy evaluations, Ipilimumab, a CTLA-4 monoclonal antibody, blocks the binding of CTLA-4 to CD80/CD86 on APCs. This action reduces the inhibitory signaling of Tregs and activates naive T cells. Consequently, it became the first immune checkpoint inhibitor (ICI) to be approved for cancer therapy due to its capacity to enhance T-cell activation and induce durable responses (26, 95).

PD-1 (programmed death 1), another crucial immune checkpoint molecule, along with its ligand PD-L1 (programmed death ligand 1), has also drawn significant attention. PD-1 and PD-L1 inhibitors enhance the immune system’s capacity to attack tumors by blocking the interaction between tumor cells and the immune system. The PD-1/PD-L1 pathway is one of the primary mechanisms of tumor immune escape. When tumor cells expressing PD-L1 bind to T cells expressing PD-1, they can relieve the inhibition of T cells, restore their killing activity, increase the proliferation of CD8+ T cells and IFN-γ secretion, and enhance cytotoxicity. The PD-1/PD-L1 pathway is expressed in over 75% of patients with uterine tumors (13). Inhibitors targeting the PD-1/PD-L1 pathway, such as nivolumab, pembrolizumab, atezolizumab, sintilimab, etc., have demonstrated excellent efficacy and safety in various tumors (96). It is important to note here that studies have shown positive PD-L1 expression in patients with HGESS tumors (25, 96, 97). Palmerini E et al. reported in the sarcoma phase 2study (NCT03277924) that, over a period exceeding 4 years among 40 DDCS cases, there was 1 complete remission (CR) and 3 partial remissions (PR). Specifically, one osteosarcoma case had a remission lasting 8.4 months, another osteosarcoma case had a 5.6 - month remission, and one Ewing sarcoma case had a 1.9 - month remission. Twenty - one cases (53%) had stable disease (SD), while 15 cases (38%) experienced disease progression (PD). However, associated hematological toxicities were observed, including leukopenia (37.5%), thrombocytopenia (37.5%), and anemia (35.0%). Among the non - hematological adverse events, the most prevalent ones were hypertension (62.5%), fatigue (62.5%), diarrhea (45.0%), and oral mucositis (45.0%) (98). In the experiment by Xie L et al., it was verified that the combined treatment of apatinib and camrelizumab extended the progression-free survival (PFS) in the treatment of advanced osteosarcoma (99). Li N et al. administered sintilimab to treat HGESS patients who had poor responses to chemotherapy after tumor recurrence following surgery, and achieved satisfactory outcomes (100). This also offers strong evidence for the future clinical use of PD-L1 inhibitors in the treatment of HGESS.

Currently, numerous studies focus on the combination of PD-1/PD-L1 inhibitors and CTLA-4 inhibitors as adjuvant therapy in conjunction with radiotherapy and chemotherapy. The aim is to eradicate residual tumor cells, prevent recurrence, and extend survival (101–109). These strategies have achieved remarkable progress in locally advanced solid tumors, including non-small cell lung cancer, head and neck cancer, and esophageal cancer. The PACIFIC study indicated that using Durvalumab, a PD-L1 inhibitor, as maintenance therapy following concurrent chemoradiotherapy increased the median overall survival (OS) of patients with stage III unresectable non-small cell lung cancer from 29.1 months to 47.5 months (110). The CALLA study also verified that the aforementioned PD-L1 inhibitor Durvalumab could significantly enhance the prognosis of patients with advanced cervical cancer treated with concurrent chemoradiotherapy (111).

ICIs exert a synergistic anti-tumor effect by reshaping the TME in multiple aspects, including releasing inhibitory signals, activating immune cells, and enhancing the physical and chemical characteristics of the microenvironment. Combined strategies targeting the TME, such as anti-angiogenesis agents and epigenetic drugs, can further enhance the efficacy of ICIs. We propose that future research on this type of HGESS should be further explored to achieve more precise and personalized treatment.

## 4 Conclusions and future directions

HGESS is a rare yet highly aggressive uterine sarcoma. Surgical resection serves as the primary treatment approach. Radiotherapy and chemotherapy can merely alleviate symptoms and fail to significantly enhance the survival rate (the median overall survival is merely 11–23 months). The recurrence rate among advanced patients is high (>70%), and they exhibit strong resistance to conventional chemotherapy. There is an urgent necessity for novel treatment methods. Immunotherapy, a groundbreaking approach, has been employed in the clinical treatment of various solid tumors. Given the breakthroughs achieved in these studies, there is good reason to believe that these immunotherapies can be applied in the clinical translation of HGESS in the future. Specifically, patients with advanced/recurrent disease, those who test positive for PD-L1, and patients carrying fusion genes may stand to benefit. Despite these encouraging results, immunotherapy still confronts some fundamental challenges that may impede its effectiveness in the clinic. It is crucial to recognize that each treatment modality has its own advantages and disadvantages (**Table 1**). This article delves into a range of immunotherapies, such as intratumoral vaccination (OVT), as well as adoptive cell transfer therapies, including TILs chimeric antigen receptor (CAR)-T cell therapy, TCR-T cell therapy, and NK therapy. Additionally, strategies like ICIs and cancer vaccines are discussed as potential avenues. The immunotherapy for HGESS remains in the early stage of exploration. Despite being supported by molecular bases (such as PD-L1 expression and fusion neoantigens) and TME characteristics (immune infiltration with strong inhibitory properties), the absence of dedicated clinical trials has significantly hindered its progress. The current focus should be placed on the following aspects: 1. Facilitate TCR-T therapy targeting fusion proteins (e.g., YWHAE-NUTM2); 2. Probe into combination regimens of immune checkpoint inhibitors (ICI) (such as anti-PD-1 + anti-CTLA-4 or oncolytic viruses); 3. Develop personalized mRNA vaccines; 4. Establish a registration research platform for HGESS immunotherapy to tackle the issue of insufficient sample size for rare diseases. With the breakthrough of AI-assisted target screening and gene editing technology, along with the future application of genetic engineering of immune cells and combination with stem cells, immunotherapy is anticipated to become the primary treatment and enter the economic era in the future.

**Table 1:** The advantages and challenges of HGESS immunotherapy.

Therapeutic regimens	Strategies	Advantages	Challenges	Solutions
**Oncolytic virus therapy (OVT)**	Genetically engineered viruses selectively infect tumor cells and release TSA to activate the immune system.	1. Dual killing: direct lysis + activation of systemic immunity. 2. Synergistic enhancement (such as in combination with ICI, CAR-T, etc.). 3. Flexible engineering.	1. Low viral delivery efficiency. 2. Weakened therapeutic effect due to antiviral immunity. 3. Lack of specific viral vector validation for HGESS.	1. Nanoparticle delivery. 2. Combined use of cyclophosphamide to suppress immunity. 3. Design of tumor microenvironment-responsive viruses.
**Cancer vaccines**	Delivery of TSAs or TAAs using mRNA/nanoparticles/viruses or cells as carriers.	1. High specificity (such as individualized neoantigens). 2. Strong immunogenicity. 3. High safety (no risk of genomic integration).	1. The new antigens of HGESS are scarce. 2. The immunosuppressive microenvironment. 3. The low efficiency of antigen presentation.	1. AI predicts new antigens (such as KRAS G12D vaccine). 2. Combined with PD-1 inhibitors. 3.Improved nanoparticle delivery system combined with mRNA vaccine
Adoptive cell transfer therapy				
TILs	Reinfusion of expanded tumor-infiltrating T cells	1. Naturally targeted individualized antigens. 2. Effective for PD-1 resistant patients (ORR 36% in melanoma).	1. TME-induced T cell exhaustion. 2. The preparation process is lengthy (> 6 weeks). 3. Difficulties in obtaining samples from HGESS.	1. Genetic engineering modification of TILs. 2. Upgrade of *in vitro* expansion technology. 3. Combination with CTLA-4 inhibitor.
CAR-T	Engineering T cells targeting surface antigens	1. The therapeutic effect of hematoma is remarkable (ORR > 80%). 2. It can be designed with dual targets (such as Claudin18.2/Occludin).	1. Heterogeneity of tumor antigens. 2. Risk of cytokine storm. 3. Immunosuppressive microenvironment.	1. Dual-target CAR-T. 2. Integration of PD-1/CD28 switch receptor. 3. Chemokine receptor modification.
TCR-T	Modifying TCR to target intracellular antigens	1. Targetable fusion protein (such as YWHAE-NUTM2). 2. High response rate for solid tumors (50% response rate for synovial sarcoma).	1. Off-target toxicity risk. 2. HLA-restricted. 3. Complex neoantigen screening.	1. AI optimizes TCR affinity 2. CRISPR eliminates endogenous TCR 3. Combined with epigenetic drugs.
CAR-NK	Engineering modification of allogeneic NK cells	1. No risk of GVHD. 2. “Ready-to-use” potential. 3. Synergistic ADCC effect.	1. Poor persistence in the body (< 1 month). 2. Insufficient infiltration of solid tumors. 3. Challenges in large-scale production.	1. Ipsc-derived NK cells. 2. Targeting the tumor stromal barrier.
**Immune Checkpoint Inhibitors (ICIs)**	Blockage of inhibitory signals such as PD-1/CTLA-4	1. 75% of uterine tumors are PD-L1 positive. 2. Prevent recurrence and prolong survival. 3. Enhance efficacy with combined radiotherapy.	1. T cell exhaustion (high TIM-3) in HGESS. 2. Immune-related adverse reactions.	1. Combined anti-angiogenic drug (bevacizumab). 2. Short-course radiotherapy sensitization.

Bold values indicate primary efficacy endpoints reported in clinical trials: • ORR, Objective Response Rate (percentage of patients with tumor shrinkage). • PFS, Progression-Free Survival (time without disease worsening). • PFS50, Proportion of patients progression-free at 6 months.

## Glossary

HGESS: High-grade endometrial stromal sarcoma
LGESS: Low-grade endometrial stromal sarcoma
TME: Tumor microenvironment
OVT: Oncolytic virotherapy
ICIs: Immune checkpoint inhibitors
PD-1: Programmed death-1
PD-L1: Programmed death-ligand 1
CTLs: Cytotoxic T lymphocytes
TCR: T-cell receptor
MHC: Major histocompatibility complex
Tregs: Regulatory T cells
MDSCs: Myeloid-derived suppressor cells
M-MDSC: Monocytic MDSC
PMN-MDSC: Polymorphonuclear MDSC
DCs: Dendritic cells
TAAs: Tumor-associated antigens
TILs: Tumor-infiltrating lymphocytes
CAR-T: Chimeric antigen receptor T cells
TCR-T: T-cell receptor-engineered T cells
NK: Natural killer cells
CTLA-4: Cytotoxic T-lymphocyte-associated antigen 4
APCs: Antigen-presenting cells
ORR: Objective response rate
OS: Overall survival
GM-CSF: Granulocyte-macrophage colony-stimulating factor
VEGF: Vascular endothelial growth factor
TGF-β: Transforming growth factor-beta
IFN-γ: Interferon-gamma
EMT: Epithelial-mesenchymal transition
